# The Syk Kinase SmTK4 of *Schistosoma mansoni* Is Involved in the Regulation of Spermatogenesis and Oogenesis

**DOI:** 10.1371/journal.ppat.1000769

**Published:** 2010-02-12

**Authors:** Svenja Beckmann, Christin Buro, Colette Dissous, Jörg Hirzmann, Christoph G. Grevelding

**Affiliations:** 1 Institute for Parasitology, Justus-Liebig-University, Giessen, Germany; 2 Inserm, U547, University Lille Nord de France, Institut Pasteur de Lille, Lille, France; Trudeau Institute, United States of America

## Abstract

The signal transduction protein SmTK4 from *Schistosoma mansoni* belongs to the family of Syk kinases. In vertebrates, Syk kinases are known to play specialized roles in signaling pathways in cells of the hematopoietic system. Although Syk kinases were identified in some invertebrates, their role in this group of animals has not yet been elucidated. Since SmTK4 is the first Syk kinase from a parasitic helminth, shown to be predominantly expressed in the testes and ovary of adult worms, we investigated its function. To unravel signaling cascades in which SmTK4 is involved, yeast two-/three-hybrid library screenings were performed with either the tandem SH2-domain, or with the linker region including the tyrosine kinase domain of SmTK4. Besides the Src kinase SmTK3 we identified a new Src kinase (SmTK6) acting upstream of SmTK4 and a MAPK-activating protein, as well as mapmodulin acting downstream. Their identities and colocalization studies pointed to a role of SmTK4 in a signaling cascade regulating the proliferation and/or differentiation of cells in the gonads of schistosomes. To confirm this decisive role we performed biochemical and molecular approaches to knock down SmTK4 combined with a novel protocol for confocal laser scanning microscopy for morphological analyses. Using the Syk kinase-specific inhibitor Piceatannol or by RNAi treatment of adult schistosomes *in vitro*, corresponding phenotypes were detected in the testes and ovary. In the *Xenopus* oocyte system it was finally confirmed that Piceatannol suppressed the activity of the catalytic kinase domain of SmTK4. Our findings demonstrate a pivotal role of SmTK4 in gametogenesis, a new function for Syk kinases in eukaryotes.

## Author Summary

Parasitic blood flukes of the genus *Schistosoma* cause schistosomiasis, one of the most important infectious diseases for humans and animals worldwide. Besides their medical importance, schistosomes possess unique biological features. Among these is the sexual maturation of the female, which requires a constant pairing contact with the male. Pairing induces mitogenic activity and differentiation processes in the female that lead to gonad development. This is a prerequisite for egg production, which is closely connected with the pathological consequences of the disease since the eggs are trapped in different host organs, inducing inflammatory processes. Although these correlations are long known, the molecular basis of differentiation processes in female gonads are poorly understood. In the context of identification of signal transduction proteins controlling female reproductive development we identified SmTK4 of *S. mansoni*, the first Syk-family kinase of a parasite. By using biochemical and molecular approaches in combination with *in vitro* culture and a novel microscopical technique, we demonstrate in this study the pivotal role of the signaling protein SmTK4 in spermatogenesis and oogenesis of *S. mansoni*. This is a new attribute for Syk kinases of eukaryotes promoting SmTK4 as a candidate target for blocking transmission and disease progression.

## Introduction

Helminth parasites of the genus *Schistosoma* are the causative agents of schistosomiasis, one of the most prevalent parasitic diseases for humans and animals worldwide [1],[2]. More than 200 million people suffer from the pathological consequences of this disease, which originate from the massive egg production of schistosomes. The eggs cause inflammatory reactions in the gut, bladder, spleen and liver leading to granuloma formation [1],[3]. Praziquantel is the only drug applicable to all schistosome species and is commonly used to treat patients, but treatment does not prevent reinfection. In the light of the absence of a vaccine and the probability of emerging resistance, a search for alternative treatments is a commonly accepted need for further research [4],[5]. In this respect great international efforts are ongoing to analyze the genome of this blood fluke, its transcriptome, proteome, and glycome [6]–[10].

Besides their medical importance, schistosomes exhibit a nearly unique biological phenomenon–the pairing-dependent induction and maintenance of the sexual maturation of the female. During a constant pairing contact, the male activates signal transduction pathways in the female leading to the proliferation and differentiation of cells in the reproductive organs, such as, the ovary and vitellarium [11]–[14]. This is a prerequisite for the female to produce about 300 eggs each day [15]. One half reaches the outside of the definitive host to deliver miracidia continuing the life cycle. The remaining eggs are deposited in the host tissue causing pathogenesis. An egg from a mature female consists of one fertilized oocyte, originating in the ovary, and 30–40 surrounding vitelline cells produced in the vitellarium. Since growth and differentiation of vitelline cells and oocytes are probably controlled by signal transduction pathways, efforts have been made to identify and characterize the participating molecules.

In the last decade, several genes encoding for signaling molecules from *S. mansoni* have been identified, some of which were found to be specifically or predominantly expressed in reproductive organs [reviewed in 16,17]. In contrast to the vitellarium, however, less is known about signaling molecules in the ovary. Among the molecules shown to be predominantly expressed in this organ is SmTK4, a member of the Syk (spleen tyrosine kinase) tyrosine-kinase family [18]. Syk kinases are characterized by a tandem Src-homology 2 (SH2) domain and a catalytic tyrosine kinase (TK) domain. Genome-project data have indicated that Syk kinase genes are absent in *Caenorhabditis elegans*, and in *Drosophila melanogaster* only the related kinase *Shark* (SH2 domain ankyrin repeat kinase; [19]) is present, which had suggested a recent evolutionary origin of kinases from the Syk family. However, Syk kinases were found in *Hydra vulgaris* as well as in sponge [20], and with SmTK4 also in the parasitic helminth *S. mansoni*.

In mammals, Syk kinases are expressed in hematopoietic cells playing well-characterized roles in inflammatory processes operating as downstream signaling molecules of immunoreceptors [21]. In the last years, evidence has accumulated for functions of Syk kinases in different signal transduction pathways also in non-hematopoietic cells [22]. Syk kinases regulate proliferation, differentiation, morphogenesis, and survival of epithelial [23],[24], endothelial [25], and neuronal cells [26]. In the hematopoietic system, Syk kinases interact with immune and antigen receptors lacking intrinsic catalytic activity [27]. The tandem-like structure of the SH2 domains confers higher binding specificity of Syk kinases to phosphorylated tyrosine residues of upstream interaction partners compared to individual SH2 domains [28]. Following receptor activation, each SH2 domain interacts with one immunoreceptor tyrosine-based activation motif (ITAM) in the intracellular part of the receptor leading to a conformational change in Syk accompanied by an increase in its enzymatic activity [29]. In SmTK4 the conserved sequence within the SH2 domains responsible for this binding is absent, suggesting that this Syk kinase interacts with molecules without ITAMs. Binding of upstream partners stimulates autophosphorylation of Syk on tyrosines within the activation loop, which influences kinase activity or creates docking sites for SH2-containing proteins [30]. The phosphorylation of Syk can be enhanced by interacting Src (Rous sarcoma virus kinase) tyrosine kinases [27]. In addition, a variety of other signaling and adaptor molecules have been reported to associate with Syk kinases, but the relevance of these interactions have not been elucidated yet [27].

With respect to the very specialized function of Syk kinases in the hematopoietic system of mammals, the existence of a schistosome homolog was unexpected. SmTK4 was found to be transcribed in the larval stages as well as adults, independently from the pairing-status. Localization studies had demonstrated its predominant expression in the testes of the male and ovary of the female, but not in the vitellarium [18]. This narrow expression profile contrasts with other identified cellular kinases in adult schistosomes, such as the Src kinases SmTK3 and SmTK5, whose expressions were demonstrated in all reproductive organs and other tissues [31],[32].

Recently, an interaction of the Src kinase SmTK3 with SmDia (Diaphanous-related formin) and SmRho1 (Ras-homologous GPTase) was shown by yeast two-hybrid (Y2H) analyses and localization studies, indicating a role of SmTK3 in cytoskeleton organization processes in the gonads of adult *S. mansoni* [33]. To elucidate the potential function of SmTK4 in adult schistosomes, two different strategies were followed in this study. First, by isolating signaling molecules acting up- and downstream of SmTK4 we expected to discover interacting proteins, whose identity could provide evidence for functionally conserved signaling pathways. Using a recently established *S. mansoni* Y2H cDNA-library of females and males [33] and different constructs of SmTK4 as probes, several interacting proteins were identified, such as SmTK3 and a novel schistosome Src kinase (SmTK6), a MAPK (mitogen-activated protein kinase)-activating protein, and mapmodulin. Second, inhibitor and RNA interference (RNAi) experiments were performed to functionally knock-down SmTK4 activity. With the Syk kinase-specific inhibitor Piceatannol [34],[35] or SmTK4-specific dsRNAs for RNAi, significant morphological changes in the testes and ovary of treated schistosomes were observed using carmine red-staining and confocal laser scanning microscopy (CLSM). In *Xenopus* oocytes it was finally shown that Piceatannol is able to suppress the catalytic activity of the TK domain of SmTK4. Taken together, our results in *S. mansoni* substantiate a pivotal role for the Syk kinase SmTK4 in gametogenesis, a function which has not yet been shown for a Syk kinase in other eukaryotes.

## Results

### Screening of a *S. mansoni* yeast two-hybrid library for upstream interaction partners of SmTK4

**Figure 1:**
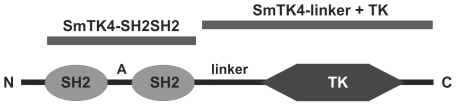
Domain structure of SmTK4. SmTK4 contains an N-terminal tandem SH2-domain (aa 48–143 and aa 201–292; A: interdomain) followed by a linker region (linker) and a C-terminally located, catalytic tyrosine kinase domain (TK, aa 881–1147). Black bars indicate the relative position of the bait constructs SmTK4-SH2SH2 (upstream screening) and SmTK4-linker+TK (downstream screening).

To identify upstream binding partners of the schistosome Syk kinase SmTK4, an adult stage Y2H cDNA-library [33] was screened with the bait construct encoding the tandem SH2-domain of SmTK4 as a fusion protein with the Gal4-BD (GAL4 DNA-binding domain) and the TK domain of SmTK3 (SmTK4-SH2SH2 + SmTK3-TK pBridge; Figure 1). The schistosome TK domain was co-expressed in a kind of yeast three-hybrid (Y3H) approach to ensure the phosphorylation of tyrosine residues of potential interaction partners, since yeast does not possess specific, endogenous tyrosine-kinase activity [36]. Phosphorylation, however, is decisive for Syk-kinase interactions, because only phosphorylated tyrosine residues of binding partners acting upstream in a signaling hierarchy are favored binding sites for the pocket-like structure of the tandem SH2-domain of Syk kinases [28],[37]. This principle has been successfully applied before to investigate the interaction between SH2 domain-containing proteins and tyrosine-containing substrates [38],[39]. Without additional protein-protein interacting domains such as SH2 or SH3, TK domains function promiscuously [40]. Therefore, we expected that tyrosine residues of yeast and library proteins were phosphorylated as a consequence of the expression of an individual TK domain. Indeed, Western-blot experiments with anti-phosphotyrosine antibodies have shown that tyrosine phosphorylation of yeast proteins was enhanced when the SmTK3 TK-domain was expressed (Phillip, unpublished). The expression of both baits (SmTK4-SH2SH2 and SmTK3-TK) was confirmed at the transcriptional level by RT-PCR analyses using total RNA extracts from transformed yeast cells (results not shown). Screening of the Y2H cDNA-library resulted in the identification of 77 initial prey clones, which underwent growth selection and β-galactosidase (β-Gal) filter assays. After isolation of the prey plasmids, sequence analyses of 14 remaining clones were performed by BlastX to unravel their identity (Table 1). Seven clones represented partial sequences from a schistosome homolog of a nonsense mRNA reducing factor (NORF1) from *Mus musculus* (accession number AAK08652). Three clones encoded proteins with homology to a dipeptidyl peptidase III from *Mus musculus* (accession number NP_598564). Two clones showed similarity to the schistosome Src/Fyn kinase SmTK5 ([32]; accession number AAF64151) and encoded a novel schistosome cellular tyrosine kinase (CTK), named SmTK6 (accession number FN397679). Finally, two clones were identical to the Src kinase SmTK3 from *S. mansoni*, identified and characterized in a previous study ([31]; accession number CAE51198).

**Table 1:** Identified upstream binding partners of SmTK4.

Clone group	Homology (accession number)	Size (bp)	E-value	Clone numbers
**A**	nonsense mRNA reducing factor NORF1 [*Mus musculus*, AAK08652]; fl: accession number CAZ33672	3978	0.0	6, 10, 19, 28, 72, 74, 75
**B**	dipetidyl peptidase III [*Mus musculus*, NP_598564]; fl: accession number CAZ29581	2034	1e^−160^	16, 37, 40
**C**	SmTK5 (Fyn/Src kinase) [*Schistosoma mansoni*, AAF64151]; fl: accession number AF232691	1944	3e^−92^	44, 45
**D**	SmTK3 (Src kinase) [*Schistosoma mansoni*, CAE51198]; fl: accession number AJ585205	1944	0.0	71, 76

Clones identified by Y3H screening using the tandem SH2-domain of SmTK4 as bait (SmTK4-SH2SH2 + SmTK3-TK pBridge). From the 77 initial prey clones obtained after library screening (mating) 14 clones resisted subsequent selection strategies, and their inserts were sequenced. BLASTX analyses showed that these clones represented four groups (A–D); a nonsense mRNA reducing factor (NORF1, group A), a dipeptidyl peptidase III (group B), a novel Src kinase (SmTK6) with similarity to the Src/Fyn kinase SmTK5 (group C), and the already known schistosome Src kinase SmTK3 (group D). To depict the e-values of the candidate clones, of which only partial sequences were obtained from the Y2H library, the full-length cDNA sequences (fl) of the clones were identified in the genome data set of the *S. mansoni* sequencing project ([6]; www.sanger.ac.uk, www.genedb.org), or in the NCBI database (www.ncbi.nlm.nih.gov) and used for BlastX analyses. The sizes of the full-length sequences, the appropriate e-values, and the yeast clone numbers are given.

### Qualitative and quantitative analyses of the SmTK4 upstream binding partners

To confirm their interactions and to determine their relative binding strength in a comparative approach, yeast cells (strain AH109) were transformed with individual prey plasmids together with the original bait construct SmTK4-SH2SH2 + SmTK3-TK pBridge. Additionally, yeast cells were transformed with the prey plasmids and the bait construct SmTK4-SH2SH2 pBridge to analyze the dependence of the observed interactions on the additional tyrosine phosphorylation. Finally, the prey plasmids were used for transformation together with bait constructs that contained only one SH2 domain of SmTK4 (SmTK4-SH2(1) or SmTK4-SH2(2) pBridge) to test if the interactions depended on the presence of the tandem SH2-domain structure. After re-transformation of the prey plasmids with the bait constructs containing the tandem SH2-domain of SmTK4, with or without the additional expression of the SmTK3 TK-domain, all yeast clones survived growth- (Trp^−^/Leu^−^/Ade^−^/His^−^) and color selection (β-Gal filter assays) thus confirming the observed interactions. However, when transformation was done with the bait plasmids containing only single SH2 domains of SmTK4, the yeast clones containing the prey plasmids encoding the Src kinases SmTK6 or SmTK3 were no longer able to grow. This indicated that Src/Syk-kinase interaction depended on the presence of the tandem SH2-domain structure. The other clones survived the selection procedure, which is a hint for unspecific interactions (Table S1).

**Figure 2:**
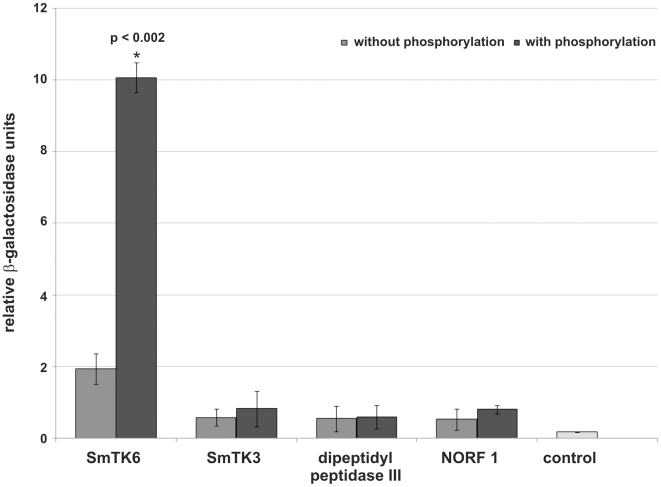
Comparative β-Gal liquid assays to determine the relative binding strengths of SmTK4 upstream interaction partners. For this analysis, yeast cells (strain AH109) were re-transformed with one representative prey clone of each group together with the bait SmTK4-SH2SH2 pBridge (light grey) or SmTK4-SH2SH2 + SmTK3-TK pBridge (dark grey). The tested clones were (from left to right): SmTK6 (Src kinase), SmTK3 (Src kinase), a homolog of a dipeptidyl peptidase III, and a homolog of a nonsense mRNA reducing factor (NORF1). As control, untransformed yeast cells were used (control). The statistical evaluation of three independent measurements of β-Gal activity (n = 3) is shown (error bars are indicated). Student's *t*-test (two-tailed): ^*^ *p*<0.002.

To quantify the relative strengths of interaction β-Gal liquid assays were performed. To this end yeast cells were used, which were transformed with representative prey plasmids encoding for each potential binding partner and the bait plasmid SmTK4-SH2SH2 pBridge, or SmTK4-SH2SH2 + SmTK3-TK pBridge. These experiments again confirmed the observed interactions with SmTK4. Furthermore, the results demonstrated the strongest interaction between the SmTK4 tandem SH2-domain and the novel Src kinase SmTK6. This interaction was increased by a factor of five when the SmTK3 TK-domain was present indicating the significant influence of additional phosphorylation (Figure 2). The interaction between the SmTK4 tandem SH2-domain and the Src kinase SmTK3 was considerably weaker, but also enhanced by additional phosphorylation, although to a minor degree. The strength of interactions between the homologs of the nonsense mRNA reducing factor (NORF1) and the dipeptidyl peptidase III were also weak and not, or only slightly, influenced by the state of phosphorylation (Figure 2).

### SmTK6 is a Src/Abl-like kinase, which co-immunoprecipitates and colocalizes with SmTK4

Since the novel Src kinase SmTK6 was the strongest upstream interaction partner of SmTK4 found in this screening, we carried out a preliminary characterization. The inserts of both SmTK6 prey clones were equal in size and about 1360 bp long. Comparisons by multiple alignment analysis revealed homology to CTKs and indicated that part of the 5′-region of the cDNA was missing in the isolated prey plasmids. Using schistosome sequencing data (www.sanger.ac.uk), the whole coding sequence was identified *in silico*. To verify its existence in our *S. mansoni* strain, we performed RT-PCR analyses using the primer pair TK6-fl-5′ (5′-CTCATTATGGGAATTTGTTTGTG-3′ containing the ATG codon) and TK6-fl-3′ (5′-AATTATCTAAATATTGAGCTTCTG-3′ containing the TAA stop codon), and total RNA from mixed-sex worms. Amplification products of the expected size were obtained and cloned. Sequence analysis showed that the complete cDNA sequence of SmTK6 is 1698 bp long encoding a protein of 566 amino acids (accession number FN397679). *In silico* analyses indicated the presence of one SH2, one SH3, and a TK domain that characterize CTKs of the Src family. Preliminary phylogenetic analyses showed that SmTK6 has also some similarity to the class of Abl kinases indicating that this kinase is a kind of Src/Abl intermediate (Beckmann, unpublished).

**Figure 3:**
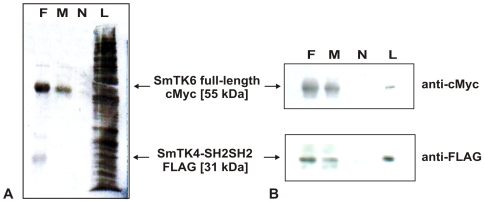
Co-immunoprecipitation of SmTK4 and SmTK6. The tandem SH2-domain of SmTK4 was expressed with a FLAG-tag, and the sequence of SmTK6 with a cMyc-tag in yeast. Co-IPs were done with lysates from yeast (strain YPH501) expressing both proteins using anti-FLAG- (F) or anti-cMyc antibodies (M). After co-IP, the proteins were separated on a SDS-PAGE and blotted onto a nitrocellulose membrane. **A:** INDIA-Ink staining of the nitrocellulose membrane (N: negative control without antibody, L: yeast lysate before immunoprecipitation). **B:** Detection of SmTK6 full-length-cMyc (55 kDa) and SmTK4-SH2SH2-FLAG (31 kDa) by Western blots using anti-cMyc or anti-FLAG-tag antibodies, respectively.

To additionally confirm the binding capacity of SmTK6 and SmTK4, co-immunoprecipitation (co-IP) experiments were performed. To this end the tandem SH2-domain of SmTK4 was cloned as a FLAG-tagged construct into one multiple cloning site of the pESC-His yeast expression vector. In the second multiple cloning site of the same vector a nearly complete version of SmTK6 was cloned as a cMyc-tagged fusion. Following yeast transformation and subsequent growth selection, protein expression was induced by galactose. The expression of the recombinant proteins was proven by Western-blot analyses (results not shown). Following co-IP, the presence of the FLAG- and cMyc-tagged schistosome proteins was finally confirmed by Western-blot analyses (Figure 3). Bands of the predicted sizes of 31 kDa (SmTK4-SH2SH2) and 55 kDa (SmTK6) were detected only in lysates precipitated by each of the antibodies, not in a control precipitated without antibody (Figure 3B). This result confirmed that the tandem SH2-domain of SmTK4 is able to bind to SmTK6 independent of additional yeast Gal4-AD/BD (GAL4 activating domain/DNA-binding domain) fusion protein partners.

**Figure 4:**
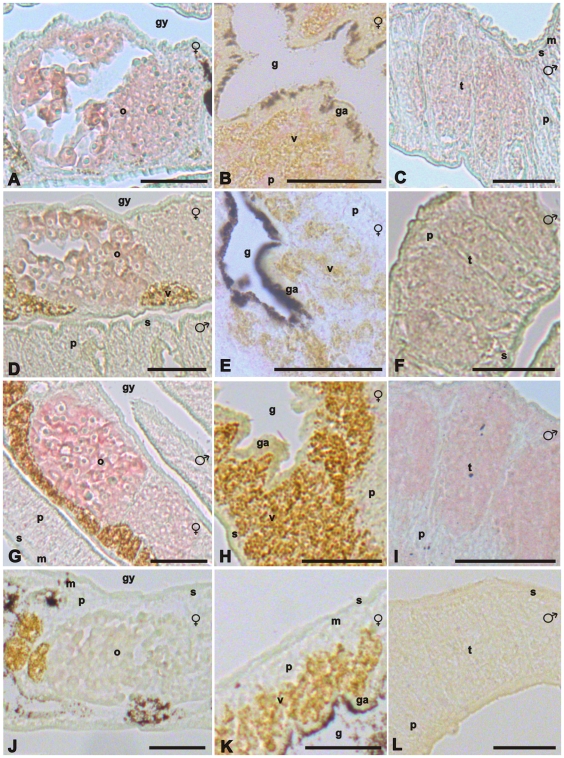
*In situ*-hybridization experiments localizing SmTK6, MAPK activating protein, and mapmodulin transcripts. Representative sections (5 µm) of adult schistosome couples (males and females are indicated), which were hybridized with DIG-labeled antisense-RNA probes of SmTK6 (A–C), MAPK-activating protein (D–F), or mapmodulin (G–I). For control, a DIG-labeled sense-RNA probe of mapmodulin was used (J–L). mRNA transcripts of all genes were detected in the ovary (o), the testes (t), and the parenchyma (p). No MAPK-activating protein gene-transcripts or mapmodulin transcripts were found in the vitellarium (v), but weak SmTK6 signals were observed in this organ. In the muscles (m), the subtegument (s), or the gastrodermis (ga), no signals were detected. [g, gut; gy, gynaecophoric canal; scale bar: 50 µm.]

By *in situ* hybridization, finally, SmTK6 was localized in the parenchyma of both genders, in the testes of the male and the ovary of the female (Figure 4, A–C). From the staining pattern obtained we cannot exclude that SmTK6 is also transcribed in the vitellarium. This testes- and ovary-preferential transcription pattern corresponded to that of SmTK4 [18], additionally supporting the conclusion that these kinases interact.

### Screening of a *S. mansoni* yeast two-hybrid library for downstream interaction partners of SmTK4

Downstream binding partners of Syk kinases are known to interact with the linker region or with the TK domain of a Syk kinase [41]. Furthermore, binding can be influenced by tyrosine residues within the TK domain, which are phosphorylated *in trans* by, e.g., Src kinases [27]. For these reasons we used SmTK4-linker+TK + SmTK3-TK pBridge as the bait construct for library screening to identify binding partners acting downstream of SmTK4 (Figure 1). This bait construct expressed the complete linker region together with the TK domain of SmTK4 fused to the Gal4-BD (multiple cloning site I, MCS I) as well as the TK domain of SmTK3 (multiple cloning site II, MCS II) to ensure tyrosine phosphorylations of bait proteins (Y3H).

The expression of the bait constructs SmTK4-linker+TK (+ SmTK3-TK) pBridge, SmTK4-linker pBridge, and SmTK4-TK pBridge was confirmed by RT-PCR analyses using total RNA extracts of the transformed yeast cells and appropriate primers (results not shown). The mating with the *S. mansoni* Y2H library resulted, after subsequent growth selection and β-Gal filter assays, in 19 clones as potential candidates for interaction. The appropriate prey plasmids were isolated and their inserts sequenced. By BlastX analyses the identities of these potential binding partners were unraveled due to their homology to proteins in the NCBI-database (Table 2). Three clones showed homology to small heat-shock proteins (HSPs) from *Caenorhabditis elegans* (accession number NP_001024376). One clone encoded a related but not identical protein, which was also homologs to small HSPs (accession number NP_001024376), as well as to the major egg antigen Smp40 from *S. mansoni* (accession number P12812). Two prey clones encoded a protein with homology to caspase 3 from *Homo sapiens* (accession number AAP36827). The inserts of five further clones encoded homologs of the MAPK-activating proteins PM20/PM21 from different organisms, such as *Homo sapiens* (accession number NP_001108072). One clone represented a leucine-rich protein from *S. mansoni* (accession number Q86QS6), which exhibited homology to mapmodulin from *Drosophila melanogaster* (accession number NP_001097361). Seven clones showed the same insert sequence encoding a protein with no significant homology to any known proteins from other organisms.

**Table 2:** Identified downstream binding partners of SmTK4.

Clone group	Homology (accession number)	Size (bp)	E-value	Clone numbers
**A**	small heat-shock protein (hsp 25) [*Caenorhabditis elegans*, NP_001024376]; fl: accession number CAZ30399	1239	5e^−06^	1, 29, 57
	Smp40 [*Schistosoma mansoni*, P12812]; fl: accession number CAZ32467; small heat-shock protein (hsp 25) [*Caenorhabditis elegans*, NP_001024376]	1404	2e^−57^; 3e^−08^	14
**B**	caspase 3 [*Homo sapiens*, AAP36827]; fl: accession number CAZ30529	891	4e^−49^	4, 7
**C**	putative MAPK-activating protein PM20/PM21 [*Homo sapiens*, NP_001108072]; fl: accession number CAZ36990	792	7e^−6^	9, 12, 13, 26, 27
**D**	leucine-rich nuclear phosphoprotein 32-related [*Schistosoma mansoni*, Q86QS6]; fl: accession number CAZ28784; mapmodulin [*Drosophila melanogaster*, NP_001097361]	660	1e^−116^; 9e^−36^	36
**E**	no significant homology			8, 22, 23, 24, 28, 35, 37

Clones identified by Y3H library screening using the linker region combined with the TK-domain of SmTK4 as bait and separately the TK domain of SmTK3 (SmTK4-linker+TK + SmTK3-TK pBridge). From the 72 initial prey clones obtained following library screening (mating) 19 clones remained positive after further selection procedures. From these clones the preys were isolated, their inserts sequenced, and after BLASTX analyses arranged into five groups (A–E) due to their homologies. The last group contains the clones showing no significant homology in the BLAST analyses (group E). The other four groups represent proteins with homology to small HSPs as well as to the schistosome major egg antigen Smp40 (group A), a caspase 3 (group B), a MAPK-activating protein (group C), and a leucine-rich protein with homology to mapmodulin (group D). To depict the e-values of the candidate clones, of which only partial sequences were obtained from the Y2H library, the full-length cDNA sequences (fl) of the clones from group A–D were identified in the genome data set of the *S. mansoni* sequencing project ([6]; www.sanger.ac.uk, www. genedb.org), or in the NCBI database (www.ncbi.nlm.nih.gov) and used for BlastX analyses. The sizes of the full-length sequences, the appropriate e-values, and the clone numbers are given.

### Qualitative and quantitative analyses of the SmTK4 downstream binding partners

**Figure 5:**
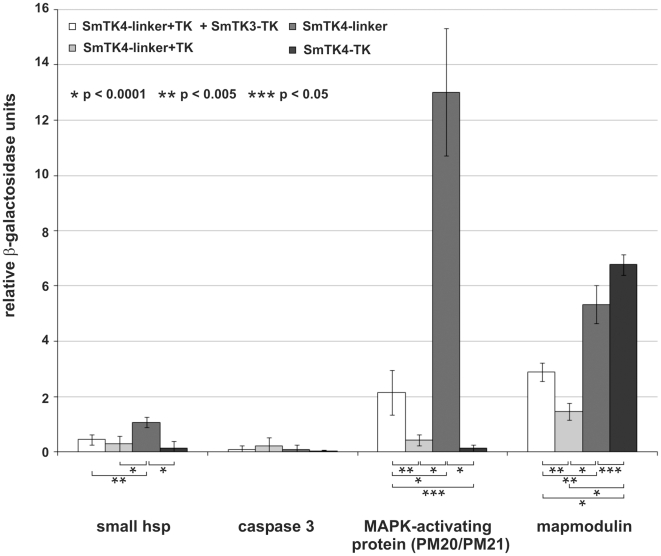
Comparative β-Gal liquid assays to determine the relative binding strengths of SmTK4 downstream interaction partners. Yeast cells (strain AH109) were re-transformed with one representative prey clone of the groups A–D together with the bait SmTK4-linker+TK + SmTK3-TK pBridge (white), SmTK4-linker+TK pBridge (light grey), SmTK4-linker pBridge (dark grey), or SmTK4-TK pBridge (black). The tested clones were (from left to right): a homolog of a small HSP 25, a caspase 3 homolog, a putative MAPK-activating protein (PM20/PM21), and a mapmodulin homolog. The statistical evaluation of six independent measurements of β-Gal activity (n = 6) is shown (error bars are indicated). Student's *t*-test (two-tailed): ^*^ *p*<0.0001, ^**^ *p*<0.005, ^***^ *p*<0.05.

β-Gal liquid assays were performed to quantify the relative binding strengths of SmTK4 and its identified downstream interaction partners. To this end yeast cells were transformed with representative prey plasmids of the groups A–D together with the original bait construct SmTK4-linker+TK + SmTK3-TK pBridge to confirm the interactions and, additionally, with SmTK4-linker+TK pBridge to analyze the dependency of interactions on the supplementary tyrosine phosphorylation. Furthermore, to determine whether the linker region and/or TK domain of SmTK4 were responsible for binding, yeast cells were transformed with individual prey plasmids and further bait constructs containing only the linker region (SmTK4-linker pBridge) or only the TK domain of SmTK4 (SmTK4-TK pBridge). The performed assays again confirmed the observed interactions with SmTK4 and, additionally, demonstrated significant differences in the relative binding strengths. The interactions between the small HSP and caspase 3 homologs with the different fragments of SmTK4 were very weak. With respect to the different SmTK4 fragments, the small HSP showed the strongest binding to the isolated linker region of SmTK4, whereas the caspase 3 homolog showed no significant differences in the relative binding strengths (Figure 5). However, the interactions of the MAPK-activating protein (PM20/PM21) and the mapmodulin with SmTK4 were stronger and differed between the SmTK4 fragments (Figure 5). In the case of the MAPK-activating protein, the interaction with the combined linker region and TK domain of SmTK4 was slightly enhanced by additional phosphorylation. However, with the isolated linker region, the interaction was nearly seven times stronger. In contrast, the interaction with the TK domain was very weak (Figure 5). These results showed that the MAPK-activating protein bound presumably to the linker region of SmTK4, and that this binding was negatively influenced by the presence of the TK domain. Furthermore, binding to the linker region was strong even without additional phosphorylation. The interaction between mapmodulin and the combined linker and TK domain of SmTK4 was also slightly enhanced by the additional phosphorylation, as in the case with the MAPK-activating protein. Regarding the isolated linker region or TK domain, mapmodulin bound strongly to both with a slight bias towards the TK domain (Figure 5). Again, interaction was strong even without additional phosphorylation. As in the case with the MAPK-activating protein, the interactions of mapmodulin with the linker region or the TK domain were negatively influenced by the presence of both domains, indicating a potential inhibitory intramolecular conformation of these domains when expressed as a fusion protein.

For the two strongest downstream binding partners of SmTK4, MAPK-activating protein and mapmodulin, colocalization with SmTK4 in the ovary of the female and testes of the male was shown by *in situ* hybridizations (Figure 4, D–I) supporting the conclusion for interaction. Corresponding to the SmTK4 transcriptional profile [18], both binding partners were found to be transcribed also in parenchyma of both genders, but not in the vitellarium of the female.

### Piceatannol treatment of adult *S. mansoni*

Recently, we introduced an experimental approach to investigate the role of schistosome CTKs by specific inhibitors. Using the Src kinase-specific inhibitor Herbimycin A for treatment of schistosome couples *in vitro*, we demonstrated that mitogenic activity and egg production were significantly reduced in females [42]. This was correlated with the reduced stability of the Src kinase SmTK3 from *S. mansoni*. Here we have combined the inhibitor approach with a novel way of phenotypic analysis at the morphological level. Based on the procedure established by Machado-Silva et al. [43] and Neves et al. [44], we investigated inhibitor effects by treatment of schistosome couples *in vitro* with subsequent fixation and carmine red-staining. By CLSM finally, we looked for morphological effects with a focus on the testes of the male and the ovary of the female, because SmTK4 expression has been detected mainly in these organs [18]. To investigate whether the inhibitors influence egg production of paired females, the numbers of eggs were counted daily during the treatment period.

Towards this end schistosome couples were treated *in vitro* with the Syk kinase-specific inhibitor Piceatannol (3,4,3′,5′-tetrahydroxy-trans-stilbene). This phenolic stilbenoid inhibits the activity of Syk kinases in cell culture with an IC_50_ value of 10 µM for the human Syk kinase [45]. For tissues it is used at higher concentrations of 100–200 µM [34].

**Figure 6:**
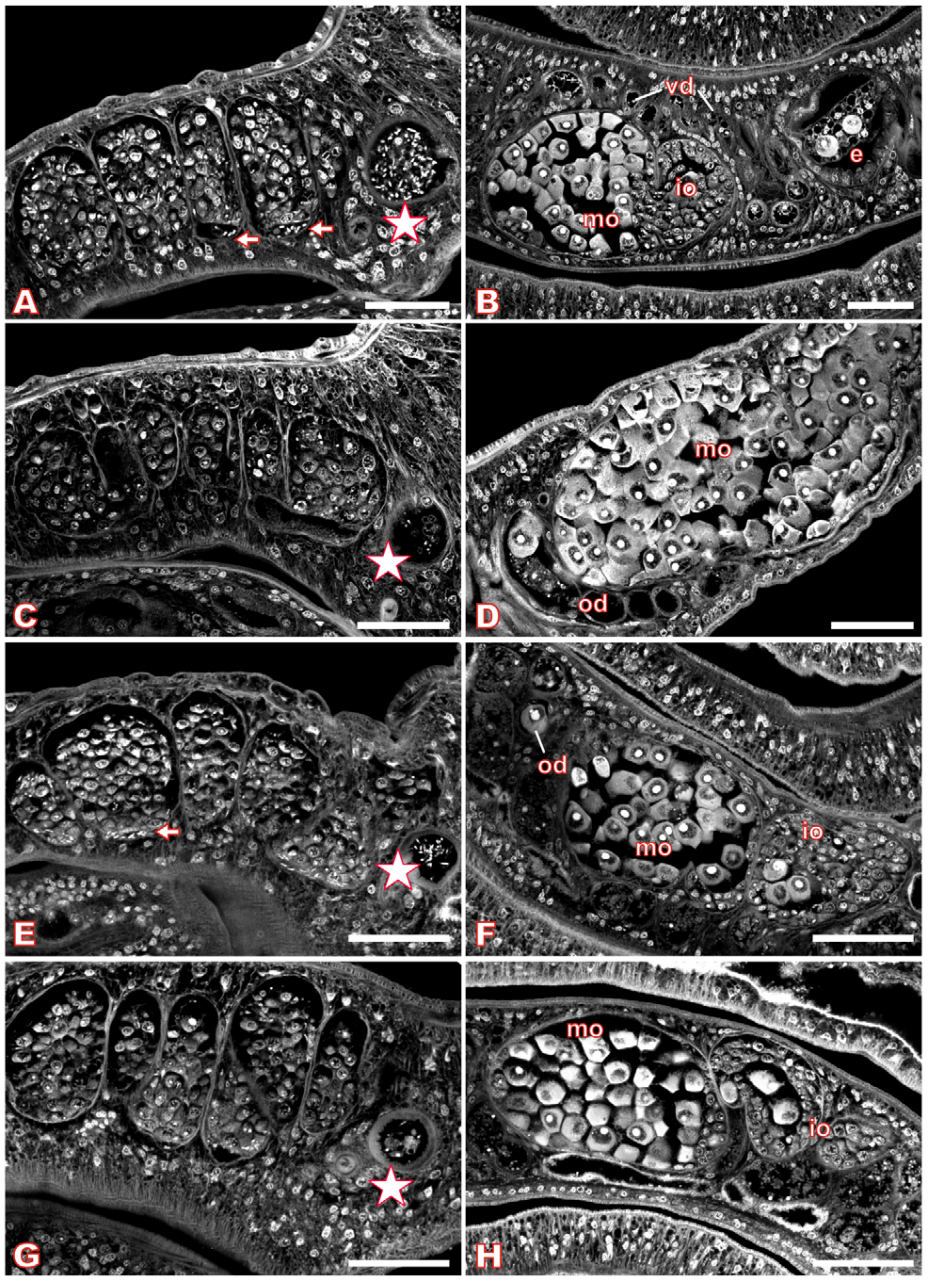
Morphology of the testes and ovary of Piceatannol- or dsRNA-treated *S. mansoni*. Confocal scanning laser microscope images of carmine red-stained whole-mount preparations of *S. mansoni* couples (A, C, E, G: males; B, D, F, H: females) treated with Piceatannol (C, D) or dsRNA (G, H). A, B: control worms treated with DMSO only; C, D: worms treated for 6 days with 70 µM Piceatannol; E, F: control worms after electroporation without dsRNA; G, H: worms after electroporation with SmTK4 dsRNA. [e: egg, io: immature oocytes, mo: mature oocytes, od: oviduct, vd: vitelloduct; asterisk: sperm vesicle, arrows: mature sperms; scale bar: 40 µm]

For *S. mansoni* couples maintained *in vitro*, we used Piceatannol at concentrations of 35 µM, 70 µM, and 100 µM over a period of six days to investigate dosage- and time-dependent effects. The medium was changed daily along with the inhibitor, and the viability of the worms as well as their pairing stability was examined. During this time period, no alterations in behavior, mortality rates, or worm pairing compared with DMSO (dimethyl sulfoxide)-treated controls could be observed (results not shown). Each day, an aliquot of treated worm couples was fixed in AFA, stained with carmine red and analyzed by CLSM. The control males and females, treated for the same time with DMSO, showed no morphological changes in the testes or ovaries (Figure 6, A–B) compared to completely untreated schistosomes ([43],[44], results not shown). The testes of untreated or DMSO-treated adult schistosome males are composed of several testicular lobes containing numerous spermatogonia and spermatocytes in different stages of maturation. Maturation of spermatocytes (spermatogenesis) begins in the dorsal part of the lobes with big round spermatogonia and ends in the ventral part with smaller elongated mature sperms (spermatozoa, Figure 6A). In the ventral part of the testicular lobes and in the *vas deferens* elongated mature sperms can be detected as well as in the anterior sperm vesicle, which is full of sperms (Figure 6A, arrows and asterisk). During treatment with 70 µM Piceatannol, however, this morphology changed considerably. After two days the size of the lobes was already slightly reduced, and the number of spermatocytes diminished (results not shown). After six days these effects were even more dramatic. The testicular lobes were shrunk accompanied by a significant decrease in the number of spermatocytes per lobe (Figure 6C). In the ventral part of the testicular lobes and in the anterior sperm vesicle, no elongated mature sperms were detected in most of the males (Figure 6C, asterisk). Instead of mature sperms, the sperm vesicle contained, in several cases, undifferentiated round spermatocytes. In addition to the effects within the testes, the inhibitor also caused morphological changes in the ovary. In untreated or DMSO-treated mature females the ovary is composed of small oogonia and immature oocytes in the anterior part and larger primary oocytes in the posterior part ([46], Figure 6B). After treatment with 70 µM Piceatannol for six days, in most of the females the number of large primary oocytes was clearly increased compared to the small, immature oocytes. Furthermore, the high number of large oocytes was distributed all over the ovary and no longer concentrated to the posterior part (Figure 6D). Using the lower concentration of 35 µM Piceatannol for worm treatment led to similar morphological changes in the testes and ovary, although with a delay of 1–2 days. A higher concentration (100 µM) led to the same phenotypes, but in a shorter time period indicating a time- and dosage-depending effect of this inhibitor on schistosomes (results not shown). Although at a comparatively low level, SmTK4 is also transcribed in subtegumental and parenchyma tissues in adults [18]. However, in these tissues we did not see an effect, which may be due to their low mitogenic activity compared to the gonads.

**Figure 7:**
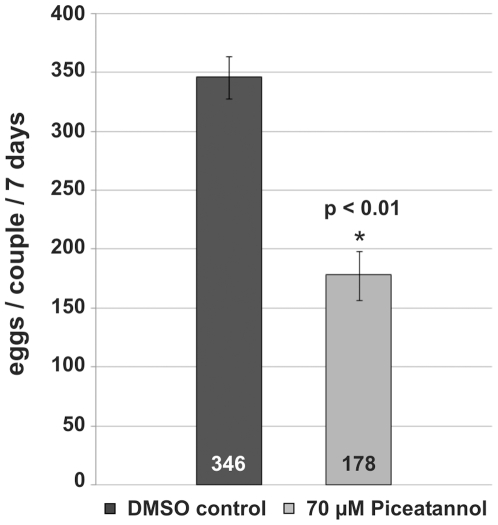
Influence of Piceatannol-treatment on egg production of *S. mansoni* couples. For this, 20 worm couples each were cultured for 7 days in the absence (DMSO control) or presence of 70 µM Piceatannol, and the egg numbers were determined at a daily basis. The statistical evaluation of four independent experiments (n = 4) is shown (error bars indicated). Student's *t*-test (two-tailed): ^*^ *p*<0.01.

To analyze the influence of Piceatannol on the egg production of paired females, the number of eggs was determined for treated couples maintained *in vitro*. 70 µM Piceatannol reduced the number of eggs per couple within 7 days to 51% compared to the DMSO control (Figure 7).

Since obvious morphological changes after Piceatannol treatment were observed, the question arose, whether these effects could be ascribed to the inhibitor's effect on SmTK4. To answer this question, evidential and experimental data were assembled pinpointing SmTK4 as the target of Piceatannol. First, Southern-blot analysis had shown that SmTK4 is a single copy gene [18], and searches through the schistosome genome data ([6], assembly version 3.1; www.sanger.uk) provided no evidence for the existence of other kinases of the Syk class. Therefore, we assume that SmTK4 is the only Syk kinase present in *S. mansoni*. Since at the concentrations used Piceatannol is specific for Syk kinases [34],[35], we concluded that SmTK4 is the only molecule targeted in *S. mansoni*. Furthermore, localization studies had indicated the testes and ovary as the only reproductive organs transcribing SmTK4, which is not transcribed in the vitellarium. In this organ, no morphological changes following inhibitor treatment were observed (result not shown).

### RNAi experiments to knock down SmTK4

To specifically attribute the effect of Piceatannol to SmTK4, we post-transcriptionally inhibited SmTK4 using dsRNAs (RNAi). To this end, SmTK4-specific dsRNAs were generated spanning both SH2 domains, including the interdomain A (linker region between the SH2 domains, Figure 1; 813 bp long). To exclude nonspecific side-effects on other tyrosine kinases, SmTK3-specific dsRNAs were also generated and applied as control. Based on the protocol from Ndegwa et al. [47], ten *S. mansoni* couples were electroporated with a single square-wave impulse and 25 µg of SmTK4- or SmTK3-specific dsRNAs, respectively. As an additional control, worm couples were electroporated under the same conditions, but without dsRNA.

To investigate the RNAi effects at the molecular level, we analyzed SmTK4 transcript levels in the three independent groups of electroporated worms by semi-quantitative RT-PCR (Figure S1). As endogenous standard, the housekeeping gene SmPDI (protein disulfide isomerase; [48]) was used. Five days after electroporation, total RNA was extracted from one half of the worm couples and the SmTK4 transcript level determined by RT-PCR analysis. The products were analyzed by agarose gel electrophoresis, and the intensities of the amplification products were densitometrically quantified using RT-PCR results of SmPDI for normalization. In three independent experiments, the SmTK4-mRNA levels were significantly reduced, although at different levels. In worms electroporated with SmTK4-dsRNA, transcript levels decreased to 10–32% compared to control worms, which had been electroporated without dsRNA. Worm couples treated with SmTK3-specific dsRNAs showed no alterations in the SmTK4-transcript level, indicating the specific silencing effect of the SmTK4-dsRNAs (Figure S1).

Finally, by carmine red-staining and CLSM the morphology of those dsRNA-treated worms was investigated, which revealed the strongest silencing effect (transcript reduction to 10%) according to RT-PCR analysis. Control worms, electroporated without dsRNA, showed no alterations in the morphology of the testes or ovary (Figure 6E–F). Additional control worms electroporated with SmTK3-specific dsRNA also showed no morphological alterations in these tissues (results not shown). However, many of the SmTK4-dsRNA electroporated worm couples exhibited phenotypes that were qualitatively comparable to the phenotypes observed after Piceatannol treatment (Figure 6G–H). In males, the size of the testicular lobes and the number of spermatocytes were reduced, and nearly no mature elongated sperms were detected in the ventral part of the lobes or within the sperm vesicle, but some immature spermatogonia (Figure 6G, asterisk). In the ovary of the female, the number of mature oocytes was increased, but not to the same extent as observed after Piceatannol treatment (Figure 6H).

### GVBD-assay in *Xenopus* oocytes to study SmTK4 activity under the influence of Piceatannol

To finally test, whether SmTK4 exerts signal transduction activities which can be suppressed by Piceatannol, we made use of the *Xenopus* oocyte system [49]. Previous studies had shown that signal transduction proteins of schistosomes can be efficiently expressed in *Xenopus* oocytes. Moreover, parasite kinase activities were already studied in this heterologous system owing to their capacity to induce resumption of meiosis or germinal vesicle breakdown (GVBD) [50],[51]. To analyze the GVBD-inducing capacity of SmTK4 and inhibitor effects on this kinase, two constructs were cloned as templates for cRNA synthesis, a full-length construct and a shortened version of SmTK4 containing only the catalytic TK domain. Following cRNA injection, GVBD was studied by the appearance of a white spot at the center of the animal pole of the oocyte. The results showed that in the absence of progesterone, a steroid inducer of GVBD [49] used for positive control, the TK domain of SmTK4 was able to induce 100% GVBD. This confirmed its catalytic activity (Table S2). In non-injected oocytes, no GVBD was observed under the same conditions as well as in oocytes transfected with the full-length variant of SmTK4. This was probably due to a close conformation of the TK domain within the complete protein. When oocytes containing the catalytic TK domain of SmTK4 were incubated with increasing concentrations of Piceatannol, however, GVBD was negatively influenced in a concentration-dependent manner and completely suppressed at a concentration of 5 µM already. The inhibitor had no effect on non-injected oocytes, and did not inhibit progesterone-dependent maturation even when it was used at the concentration of 100 µM (Table S2). These results confirmed the specific action of Piceatannol on SmTK4.

## Discussion

SmTK4 was the first Syk kinase identified in a parasitic helminth and shown to be predominantly expressed in the testes and ovary of adults [18]. To elucidate signaling cascades in which SmTK4 participates, screenings of a *S. mansoni* adult stage Y2H library were performed. Using the tandem SH2-domain as bait, upstream binding partners of SmTK4 were identified such as homologs of a nonsense mRNA reducing factor, a dipeptidyl peptidase III, SmTK3, and SmTK6. Although most clones represented homologs of a nonsense mRNA-reducing protein factor (group A, [52]), binding tests with individual SH2 domains failed, indicating nonspecific interaction. Furthermore, interactions between Syk kinases and such factors are not known. Both arguments also apply to dipeptidyl peptidase III (group B, [53]), which represents another nonspecific interaction partner. The other two upstream interaction partners belong to the Src family of CTKs (groups C, D). One represented the already characterized SmTK3 [31], whilst the other was a novel Src kinase named SmTK6. In contrast to group A and B clones, the binding of both Src kinases occurred only with the tandem SH2-domain of SmTK4, and was enhanced by additional phosphorylation in yeast, in the case of SmTK6 by a factor of five. This confirmed the specificity of the detected Syk-Src interactions.

SmTK6 turned out to be the strongest upstream interaction partner, and *in situ* hybridizations demonstrated its transcription within the testes of the male and the ovary of the female. This corresponds to the transcription profiles of SmTK4, but also SmTK3, which among other tissues is also expressed in the testes and ovary [31]. In addition to the interaction analyses, the colocalization of SmTK6, SmTK3 and SmTK4 allows the conclusion that these kinases cooperate in the gonads. Src-Syk interactions are already known from other systems. In mammalian immune cells, Src kinases recruit downstream-acting Syk kinases to the plasma membrane to activate them by phosphorylation [54],[55]. Thus, high-affinity binding sites are created for molecules acting downstream of Syk [27] that bind to phosphotyrosine residues within the linker region, or the TK domain of Syk [27],[41],[56]. From these data we hypothesize that SmTK3 may recruit SmTK6 to the plasma membrane, where the latter one becomes phosphorylated. This may be a prerequisite for binding of SmTK4 by its SH2 domain. By co-IP we found further evidence for SmTK4-SmTK6 binding, which supports the sketched scenario. Future studies will aim to analyze these interactions in more detail.

As downstream interaction partners of SmTK4 homologs of caspase 3, small HSP, a PM20/PM21 type MAPK-activating protein, mapmodulin, and a protein with non-significant similarity were found. The caspase 3 homolog (group B, [57]) showed the weakest relative binding strength with all SmTK4 subfragments. Although for ZAP-70, the second member of the Syk-kinase family in mammals, an influence on caspase pathways has been described [58], this influence is indirect [59], and no direct interaction between Syk kinases and caspases has been described yet. In light of these facts, a nonspecific binding may have occurred. The relative binding strength of the small HSP homolog (group A) was slightly stronger as for caspase 3. Besides other functions [60], HSPs fulfill chaperone functions, and a protective role of HSP90 for ZAP-70 has been demonstrated [61]. Therefore, we assume that the schistosome HSP homolog may fulfill a chaperone-like function for SmTK4. Mapmodulin (group D), a microtubule-associated protein [62],[63], showed high relative binding strengths to both the linker region and the TK domain indicating that it is able to bind to both fragments of SmTK4. This suggests that SmTK4 may influence the reorganization of the cytoskeleton in spermatocytes or oocytes, which is supported by the *in situ*-hybridization data showing mapmodulin transcripts in the testes and ovary. Indeed, Syk kinases are known to phosphorylate substrates involved in cytoskeleton organization, or components of the cytoskeleton such as microtubules directly [23],[64],[65]. Finally, the MAPK-activating protein of the PM20/PM21 type (group C) showed the strongest relative binding activity to SmTK4. Since this group of proteins lacks defined structural or functional domains, the molecular basis for interaction as well as the function of the schistosome MAPK-activating protein remains uncertain [66],[67]. Results of our study provide first evidence for its binding to the linker region of SmTK4. MAPK signaling is acknowledged to be initiated by upstream molecules, such as growth factors and RTKs, which are known to be components of signaling cascades controlling proliferation, differentiation, and survival of cells. A critical role of Syk for MAPK activation has been postulated [68], although no direct interactions between Syk kinases and a MAPK-activating protein were shown yet. *In situ*-hybridization experiments colocalized transcripts of the schistosome MAPK-activating protein in the testes and ovary providing further evidence for interaction. This allows the speculation that SmTK4 may trigger a MAPK signaling-pathway in the gonads probably influencing cell proliferation. We tried to find evidence for MAPK activation using Western-blot analyses with antibodies directed against phosphorylated or non-phosphorylated forms of human MAP kinases and protein homogenates from mixed sex schistosomes. In another independent study, these antibodies showed a high specificity also for *Echinococcus* MAP kinases [69]. Although a highly conserved MAPK homolog exists in the genome of *S. mansoni* ([6], accession number XP_002575049), its structure may be different since the antibodies were not able to detect a band of the expected size (results not shown). However, the capacity of the TK domain of SmTK4 to induce GVBD in *Xenopus* oocytes provided at least indirect evidence for the potential of the schistosome Syk kinase to activate a MAP kinase cascade since GVBD is only induced when this signaling pathway has been activated [70].

The differentiation of germ cells in vertebrates and invertebrates depends on the re-organization of the actin and tubulin cytoskeletons [71]–[73]. The participation of Syk kinases in these processes, either directly by phosphorylation of cytoskeleton components [64], or indirectly by the activation of further signaling molecules has already been described [74],[75]. The identity of the isolated up- and downstream interaction partners of SmTK4 allowed first speculations for a role of SmTK4 in signaling pathways, regulating the proliferation and/or differentiation of germinal cells by activation of a MAPK pathway and/or by influencing cytoskeleton rearrangements. To investigate a cytoskeletal role, the Syk kinase-specific inhibitor Piceatannol was used for functional inhibition of the protein and second, SmTK4-specific dsRNAs were applied for post-transcriptional gene silencing. Since Southern-blot [18] and *in silico* analyses confirmed that SmTK4 is the only Syk kinase in *S. mansoni*, and since at the concentrations used Piceatannol only inhibits Syk kinases [34],[45], the morphological effects observed by CLSM could be specifically attributed to an inhibition of SmTK4. This conclusion was supported by GVBD assays in *Xenopus* oocytes confirming that Piceatannol is able to specifically inhibit the catalytic activity of the TK domain of SmTK4 in a concentration-dependent manner. Inhibitor and dsRNA treatment seemed to disrupt sperm development in schistosome males at an early stage, since the number of mature sperms as well as of spermatocytes was reduced within the testicular lobes. It seems likely that the inhibition influenced the proliferation of the spermatogonial cells in the dorsal part of the testes. The proliferation of these cells is necessary for the initiation of spermatogenesis and the continuous production of mature sperms [76]. Thus, a dysfunction of spermatogenesis at this early stage would lead to the absence of mature sperms. This coincides with studies from other organisms (mouse, human) showing that the proliferation of spermatogonial cells is activated by CTKs. Accordingly, Src-specific inhibitors or RNAi led to dysfunctions in spermatogenesis [76]. For CTKs of the Src family, an involvement in spermatogenesis is well-known, and this has also been hypothesized for the Lyn- and Fyn-family kinases [76],[77]. Indeed, the CLSM analysis of *S. mansoni* couples treated with the Src kinase-specific inhibitor Herbimycin A also showed significantly reduced numbers of sperms in the ventral part of the testicular lobes and within the sperm vesicle [78]. It was shown before that Herbimycin A affects the biochemical activity of SmTK3 [31], but we cannot exclude that it also affects SmTK6 activity. In contrast to the Src-family kinases, however, the involvement of a Syk kinase in spermatogenesis has not been confirmed previously in eukaryotes.

Harayama et al. [79] first reported the existence of a Syk kinase in spermatozoa of mammals. Here Syk is phosphorylated and activated by a cAMP-activated PKA indicating the involvement of a signaling pathway, which differs from that in hematopoietic cells. It was speculated that the PKA-Syk pathway is associated with the fertilization capacity of sperms [79], but functional data have not been provided yet. In untreated schistosome females the anterior part of the ovary contains the immature developing oogonia which have stem-cell character, whereas the posterior part contains mature primary oocytes with enlarged cytoplasms. As in other trematodes, primary oocytes leave the ovary to become fertilized within the oviduct before entering meiosis [46],[80]. Upon Piceatannol treatment, an increased number of primary oocytes and fewer immature oocytes were observed in females. One explanation for this structural change is an inhibition of processes occurring early in oocyte development such as oogonial divisions, leading to a reduced supply of primary oocytes. This is similar to the phenotype observed in the testes and supported by the result of the egg-count reduction test, which showed a significant reduction of egg production within one week in paired females treated with Piceatannol. We expected a complete fading of egg production with elongated time of treatment. However, a long-term effect of Piceatannol on egg production could not be studied because worms died nearly 10 days after treatment in culture before egg production had terminated. Furthermore, a hatching assay showed no morphological differences between developing eggs of inhibitor-treated worms or controls, indicating that SmTK4 may not have an essential function for egg maturation or developing miracidia (results not shown). RNAi approaches performed to post-transcriptionally silence SmTK4 resulted in similar phenotypes in the testes and ovary of adult *S. mansoni*. This indicates that electroporation combined with soaking was suitable and efficient to induce RNA silencing in the gonads. In previous studies, RNAi effects were observed for genes expressed in the tegument or in the gastrodermis [47],[81], tissues more easily accessible to dsRNA. At this time-point, the RNAi protocol for adult schistosomes is not fully reproducible in our hands, as indicated by variable rates of SmTK4 knock-down in the three independent experiments, and by the absence of a phenotype in the SmTK3-dsRNA control. Since the SmTK4-dsRNAs reduced the transcript levels incompletely, phenotypes had been obtained that were qualitatively but not quantitatively comparable to the Piceatannol-induced phenotypes. The efficiency of this inhibitor to suppress SmTK4 function was higher resulting in more dramatic phenotypes in the gonads of adult schistosomes.

In oocytes of marine nemertean worms (*Cerebratulus* sp.), inhibitor studies also showed evidence for a role of Syk kinases, as well as of Src-like CTKs during oocyte maturation [82]. Using the nonspecific tyrosine kinase inhibitor genestein (50–100 µM), or the Syk-kinase specific inhibitor Piceatannol (50–100 µM) a decrease in the maturation of oocytes was observed, indicated by reduced GVBD occurring in early meiosis, and also by reduced MAPK activity. With Src-kinase inhibitors (100 µM PP2, 20 µM SU6656) no reduction in GVBD was observed, but in that of MAPK activity. Based on their data Stricker & Smythe [82] propose that in oocytes of nemertean worms the signal is transduced from RTKs to Syk kinases, followed by Src kinases and ending in a MAPK-signaling pathway that leads to the activation of MPF (maturation-promoting factor) and GVBD. This model is basically, but not completely supported by the findings of our study. Besides the indirect evidence for the MAP kinase cascade-inducing capacity of SmTK4 in *Xenopus* oocytes, Y2/3H analyses identified its interactions with the Src kinases SmTK6 and SmTK3 as upstream partners as well as with a MAPK-activating protein as one of two candidate downstream partner. Since Src kinases are activated by RTKs, it seems feasible to us that in *S. mansoni* oocytes the signal is transduced from a RTK to a Src kinase leading to the activation of the Syk kinase SmTK4, which in turn activates a MAPK cascade.

Its essential function for gonad development and the long-term killing effect of worms by Piceatannol in culture suggest that SmTK4 may be a candidate target for blocking transmission and disease progression. Although CTKs such as Syk kinases are conserved in the animal kingdom, there may be possibilities to target specific homologs. A comparison of human Syk (accession number AAH11399) and SmTK4 (accession number CAD13249) revealed differences at the amino acid level between 17% (within the catalytic domain) and 63% (whole protein). This and the remarkable elongated linker-domain region of SmTK4 may provide a basis for selective drug design.

## Materials and Methods

### Ethics statement

All experiments involving hamsters within this study have been performed in accordance with the European Convention for the Protection of Vertebrate Animals used for Experimental and other Scientific Purposes (ETS No 123; revised Appendix A) and have been approved by the Regional Council (Regierungspraesidium) Giessen (V54-19 c 20/15 c GI 18/10).

### Yeast two-/three-hybrid screening

A Y2H cDNA-library based on RNA of adult male and female *S. mansoni* was constructed [33]. To this end, cDNAs were cloned into the prey vector pGADT7-Rec (leucine nutritional marker LEU2, Clontech) in frame with the GAL4 activation domain (Gal4-AD). For subsequent library screening, mating was performed according to the user manual (Yeast protocols handbook, Clontech). Two yeast strains were used for the mating; the library-containing strain AH109 (Mat a; reporter genes ADE2, HIS3, and LacZ) and the bait-containing strain Y187 (Mat α; reporter genes HIS3, LacZ). As bait vector for library screening, the plasmid pBridge (tryptophan nutritional marker TRP1, Clontech) was used, which contains two multiple cloning sites, MCS I and MCS II. MCS I allows the cloning of protein-coding gene sequences as fusion constructs with the GAL4 DNA binding domain (Gal4-BD). MCS II permits the cloning of a second gene sequence for the expression of an additional protein and, therefore, allows the establishment of a Y3H system.

### Cloning of the bait vectors

Screening for upstream interaction partners of SmTK4 was performed with a bait vector containing the tandem SH2-domain of SmTK4 cloned into the MCS I in frame with the GAL4 DNA-binding domain (Gal4-BD). The encoding sequence was amplified by PCR using the SmTK4-specific primer pair SmTK4-SH2SH2-5′ (5′-GGATCCGTGGAGCTATTCCAC-3′; containing a *Bam*HI restriction site) and SmTK4-SH2SH2-3′ (5′-CTGCAGTGATATACCACCGGA-3′; containing a *Pst*I restriction site), and a full-length cDNA clone of SmTK4 as template. Amplification products of the expected size were cloned *via Bam*HI/*Pst*I into the MCS I of the vector pBridge. After cloning, the resulting construct SmTK4-SH2SH2 pBridge was sequenced to confirm the correct open reading frame of the Gal4-BD/SmTK4-SH2SH2 fusion. To perform a Y3H screening for upstream binding partners of SmTK4 a second bait vector was constructed, containing SmTK4-SH2SH2 in the MCS I and, additionally, the coding sequence for the TK domain of the schistosome Src kinase SmTK3 in the MCS II. To this end, the sequence for the SmTK3 TK-domain was amplified by PCR using the SmTK3-specific primers SmTK3-TK-5′ (5′-GCGGCCGCATCATCCAGAACCTGTGGG-3′; containing a *Bgl*II restriction site) and SmTK3-TK-3′ (5′-AGATCTGCTGGTTGCTCATCTTCAC-3′; containing a *Not*I restriction site), and a full-length cDNA clone of SmTK3 as template. The PCR product was cloned *via Bgl*II/*Not*I into the MCS II of SmTK4-SH2SH2 pBridge resulting in the construct SmTK4-SH2SH2 + SmTK3-TK pBridge. The success of this cloning approach was confirmed by sequencing. For control studies to test binding specificities, modified versions of this construct were additionally cloned by deletion of either the N-terminal or the C-terminal SH2-domain of SmTK4 (SmTK4-SH2(1) + SmTK3-TK pBridge, SmTK4-SH2(2) + SmTK3-TK pBridge).

To screen for downstream interaction partners of SmTK4 three different bait vectors were constructed, containing either the linker-region of SmTK4, the linker-region and the TK domain, or only the TK domain of SmTK4 in the MCS I. The coding sequences of these regions of SmTK4 were amplified by PCRs with the primer pairs TK4-bait1-5′ (5′-GGATCCTACAGAAACCAATACCAGTATC-3′) + TK4-bait1-3′ (5′-CTGCAGGGTATGCAAATATTTGTTTGT-3′), TK4-bait1-5′ + TK4-bait2-3′ (5′-CTGCAGTTCAACAAGAAATTCGATG-3′), or TK4-bait2-5′ (5′-GGATCCAAATTTATGATGAATTACCACC-3′) + TK4-bait2-3′, respectively, and a full-length cDNA clone of SmTK4 as template. The 5′-primers contained a *Bam*HI restriction site and the 3′-primers a *Pst*I restriction site, respectively. Amplification products of the expected sizes were cloned *via Bam*HI/*Pst*I in the MCS I of pBridge in frame with the Gal4-BD, resulting in the constructs SmTK4-linker+TK pBridge, SmTK4-linker pBridge, and SmTK4-TK pBridge. For Y3H analysis, an additional vector was constructed, containing the SmTK4 linker region and TK domain in the MCS I, and the SmTK3 TK-domain in the MCS II (SmTK4-linker+TK + SmTK3-TK pBridge). The integrity of the open reading frames with the Gal4-BD was confirmed by sequencing.

### Screening of the yeast two-hybrid library

The screening for upstream or downstream interaction partners of SmTK4 was performed with either the bait construct SmTK4-SH2SH2 + SmTK3-TK pBridge or SmTK4-linker+TK + SmTK3-TK pBridge, respectively. Yeast cells (strain Y187) were individually transformed with both plasmids by the lithium acetate method (Yeast protocols handbook, Clontech). For the screening of the *S. mansoni* library, bait-expressing Y187 cells were mated with library-containing AH109 cells. Mating efficiencies of 75% or 28% were obtained, respectively, which exceeded the required minimum of 2% in both cases (Clontech). The first selection of diploid yeast cells containing interacting proteins was carried out on synthetic dropout medium lacking the amino acids tryptophan, leucine, and histidine (Trp^−^/Leu^−^/His^−^). To enhance the selection pressure on clones with interacting proteins, grown colonies were plated onto synthetic dropout medium lacking the amino acids tryptophan, leucine, histidine, and adenine (Trp^−^/Leu^−^/His^−^/Ade^−^). For further selection, β-Gal colony filter assays were performed using X-Gal as substrate according to the manufacturer's instructions (Yeast protocols handbook, Clontech). From positively tested yeast clones, plasmid DNA was isolated using cell disruption by vortexing with glass beads (Sigma) followed by plasmid preparation (PeqLab). Isolated plasmid DNA was transformed into heat shock-competent *Escherichia coli* cells (DH5α), and the bacteria selected on LB-plates containing ampicillin (100 µg/µl). To differentiate bacterial colonies containing the pBridge bait-plasmid from those containing a pGADT7 prey-plasmid, colony PCRs with pGADT7-specific primers were performed. Prey plasmids from PCR-positive bacterial clones were isolated and sequenced commercially (AGOWA, Berlin). For further binding analyses, the yeast strain AH109 was transformed with appropriate prey plasmids together with different bait plasmids. To confirm protein-protein interactions, the selection procedures were repeated. For quantification of relative interaction strengths, β-Gal liquid assays with ONPG (o-nitrophenol-galactopyranoside, SIGMA) as substrate were performed according to the Yeast protocols handbook from Clontech.

### Isolation of RNA and proteins from yeast

For isolation of yeast total RNA, a 5 ml overnight culture of the appropriate yeast clone was centrifuged to harvest cells. The pellet was washed two times with PBS and afterwards frozen in liquid nitrogen. Cells were disrupted by three freeze/thaw cycles (liquid nitrogen, 37°C water bath), 1 ml TriFast (PeqLab) was then added to the cell lysate, and total RNA was extracted according to the manufacturer's instructions.

Total protein extracts from yeast cells were obtained using the urea/SDS method as described in the Yeast protocols handbook (Clontech). For the inhibition of endogenous proteases and phosphatases, the buffer for cell disruption was supplemented with PMSF (phenylmethylsulfonylfluoride, SIGMA), protease inhibitor cocktail (Complete Mini, Roche), NaF (sodium fluoride, 50 mM), and Na_3_VO_4_ (sodium orthovanadate, 2 mM). Protein extracts were analyzed by standard SDS-PAGE and Western blotting with an anti-phosphotyrosine-specific antibody (Santa Cruz Biotechnology).

### RT-PCR analyses of yeast transcripts

To confirm the transcription of the different bait-constructs inside the yeasts, RT-PCRs were performed with total RNA from bait-containing yeast clones as template. RT-PCRs were carried out stepwise in two separate reactions. First, 90 ng of total RNA was converted into cDNA using the SensiScript reverse transcriptase (Qiagen) and oligo-d(T), or bait sequence-specific primers. One fourth of the RT reaction volume was used for PCR amplification using appropriate bait sequence-specific primers. Amplification products were analyzed by agarose gel electrophoresis.

### Co-immunoprecipitation

The pESC-His yeast expression system (Novagen) was used for immunoprecipitation experiments. The pESC-His vector contains two multiple cloning sites (MCS I, contains a FLAG-tag sequence; MCS II contains a cMyc-tag sequence) under the control of galactose-inducible GAL10 or GAL1 promoters. The tandem SH2-domain of SmTK4 was cloned into MCS I (FLAG-tag at the C-terminus), and the nearly complete version of SmTK6 (except 250 bp of the N-terminus) was cloned into MCS II (cMyc-tag at the N-terminus). For cloning, the tandem SH2-domain of SmTK4 was amplified by PCR using gene-specific primers containing appropriate restriction sites (TK4-SH2SH2-pESC-His-5′(*Not*I): 5′-GCGGCCGCAATGGGAGCTATTCCACCG-3′; TK4-SH2SH2-pESC-His-3′(*Cla*I): 5′-ATCGATGATATACCACCGGAACCTGA-3′). Before cloning into MCS II, the SmTK6 sequence was also PCR-amplified using gene-specific primers with restriction sites (TK6-Voll-pESC-His-5′(*Xho*I): 5′-CTCGAGAATGTTGTGACTGATGTGCAT-3′; TK6-Voll-pESC-His-3′(*Sac*II): 5′-CCGCGGTTATCTAAATATTGAGCTTCTGTGTGC-3′). The integrity of the cloned constructs was confirmed by sequencing (AGOWA, Germany).

Following transformation, yeast cells (strain YPH501) were grown for 5 days at 30°C on selection media (synthetic drop-out medium [SD], His^−^, + glucose) to ensure the presence of the vector. Selected clones were grown over night in SD (His^−^, + glucose) liquid medium. The cells were centrifuged the next day and resuspendend in SG medium (His^−^, + galactose) and incubated for 5 h to induce transgene expression. As control, transformed yeast cells were alternatively resuspendend in SD (His^−^, + glucose). Protein was isolated from 10 ml culture volume for electrophoresis (10 µg protein each; 10% SDS-PAGE). After blotting on nitrocellulose (Schleicher & Schüll) the induction of the expression of the recombinant schistosome proteins was confirmed by Western-blot analysis using an anti-FLAG-tag (2.5 µg/µl; Novagen) or an anti-cMyc-tag antibodies (1 µg/µl; Novagen). A goat anti-rabbit-HRP (horse raddish peroxidase) secondary antibody was used for detection (1∶70.000; Novagen). Individual bands of the expected sizes of 31 kDa (SmTK4-SH2SH2) or 55 kDa (SmTK6) were observed in proteins obtained from the galactose-induced yeast culture but not in proteins obtained from the glucose-induced culture (results not shown).

For co-IP 70 µl of the protein lysate of the galactose-induced culture were pre-incubated with 30 µl protein A sepharose (Sigma) to remove proteins binding unspecifically. The remaining lysate was splitted in two parts, and each half incubated (over night at 4°C) with 4 µg anti-FLAG-tag antibody or 1 µg anti-cMyc-tag antibody, respectively. Antibody complexes were precipitated by protein A sepharose (2 h at 4°C). Following washing steps, the protein complexes were eluated from the protein A sepharose, and a Western-blot analysis was done with the recovered yeast protein as described above. After nitrocellulose transfer, the proteins were visualized by INDIA ink staining (Pelikan, Germany; 1% acetic acid, 0.04% Tween 20, 0.1% Fount India).

### Transcription analyses of schistosome genes

To monitor the transcription of SmTK4 in control and treated *S. mansoni*, total RNA was extracted using TriFast (PeqLab) following the manufacturer's instructions. Residual DNA remaining in the RNA preparations was removed by DNase digestion using RNase-free DNaseI (Fermentas). cDNA was synthesized using 1 µg total RNA, 1 µl oligo-d(T) primer (dT_24_VN; 20 µM), 1 µl nonamer primer (dN_9_; 20 µM) and Superscript II reverse transcriptase (Invitrogen). Subsequent PCRs were performed with 1/10 of the cDNA as template, FIREPol *Taq* polymerase (Solis BioDyne), and the following primer combination: SmTK4-Sub3-5′ (5′-ATGACGTAAAAGATTCACGTG-3′) and SmTK4-Sub3-3′ (5′-TGCATGTTCTTCACTACAATC-3′), which flank the region used as target for the dsRNAs. For normalization, the transcription of the housekeeping gene SmPDI [48] was monitored using the same cDNAs as template, but using the following primer combination: SmPDI-5′ (5′-GGGATTTATCAAGGATACGGACTC-3′) and SmPDI-3′ (5′-CACCAAGGAGCATACAGTTTGAC-3′). All PCRs were performed in a final volume of 25 µl. PCR products were separated on 1.5% agarose gels stained with ethidium bromide. The relative intensities of the amplification products were determined densitometrically using the program ImageJ (version 1.4.1; http://rsbweb.nih.gov/ij/index.html). For relative quantification of the SmTK4 products, the SmPDI products were used as endogenous standard.

*In situ* hybridizations were done as described elsewhere in detail [31],[33]. In short, adult worm pairs were fixed in Bouin's solution (picric acid/acetic acid/formaldehyde; 15/1/5) before embedding in paraplast (Histowax, Reichert-Jung). Sections of 5 µm were generated and incubated in xylol to remove the paraplast. Following re-hydration, proteins were removed by proteinase K treatment (final concentration 1 µg/ml), and the sections were dehydrated. For hybridization, *in vitro* transcripts were labeled with digoxigenin following the manufacturers' instructions (Roche). Labeled sense and antisense transcripts of SmTK6 (unique site, length 354 bp), the MAPK activating protein PM20/21 (length 452 bp), or mapmodulin (length 420 bp), were size-controlled by gel electrophoresis. To prove their quality, transcript blots were made to confirm digoxigenin incorporation by alkaline phosphatase-conjugated anti-digoxigenin antibodies, naphthol-AS-phosphate, and Fast Red TR (Sigma). All *in situ* hybridization were performed for 16 h at 42°C. Sections were stringently washed up to 0.5×SSC, and detection was achieved as described for transcript blots.

### Parasite stock

A Liberian isolate of *S. mansoni* was maintained in *Biomphalaria glabrata* as intermediate host and in Syrian hamsters (*Mesocricetus auratus*) as definitive host [83]. Adult worms were obtained by hepatoportal perfusion at 42–49 days post-infection.

### *In vitro* culture of adult schistosomes

After perfusion, adult schistosomes were washed three times with M199 medium before being cultured *in vitro* in M199 (Gibco; including glucose, sodium bicarbonate, 4-(2-hydroxyethyl)-1-piperazineethane sulfonic acid) supplemented with an antibiotic/antimycotic mixture (1.25%, Sigma) and FCS (10%, Gibco) at 37°C and 5% CO_2_ [42]. For each experiment, 10–30 pairs of *S. mansoni* were kept in 60 mm diameter culture dishes in 3 ml culture medium. The medium was changed every 24 hours. If needed, schistosome pairs were carefully separated with fine tweezers.

### Inhibitor treatment and morphological analysis

Piceatannol (3,4,3′,5′-Tetrahydroxy-trans-stilbene; Alexis Biochemicals) was dissolved in dimethyl sulfoxide (DMSO) (5 µg/µl). For each inhibitor treatment experiment 20 adult couples of *S. mansoni* were maintained in 10 ml culture medium [42], supplemented with various concentrations of Piceatannol (35 µM, 70 µM, 100 µM). Medium and inhibitor were refreshed every 24 hours during the treatment periods *in vitro*. For morphological analysis, adult worms were fixed for at least 24 hours in AFA (alcohol 95%, formalin 3%, and glacial acetic acid 2%), stained for 30 minutes with 2.5% hydrochloric carmine (Certistain®, Merck), and destained in acidic 70% ethanol. After dehydration for 5 minutes in 70%, 90%, and 100% ethanol, respectively, worms were preserved as whole-mounts in Canada balsam (Merck) on glass slides [43],[44]. CLSM images were made on a Leica TSC SP2 microscope using a 488 nm He/Ne laser and a 470 nm long-pass filter in reflection mode.

### RNAi experiments

As basis for double-stranded RNA (dsRNA) synthesis, a 813 bp fragment of the SmTK4-coding DNA was amplified by PCR using the gene-specific primers SmTK4-5′ (5′-ATGCCTGGAGCTATTCCA-3′) and SmTK4-3′ (5′-TGATATACCACCGGA-3′) and a full-length cDNA clone of SmTK4 as template. The amplification product of expected size was cloned into the pDrive cloning vector (Qiagen). The resulting construct, containing T7 and SP6 RNA polymerase promoters flanking the SmTK4 sequence was used to generate single-stranded RNA by *in vitro* transcription with T7 and SP6 RNA polymerases (MEGAscript RNA transcription kit, Ambion). The single-stranded RNAs were purified by LiCl-precipitation, resuspended in dH_2_O, and quantified by spectrophotometry. Equal amounts of the single-stranded RNAs were mixed in annealing buffer (500 mM potassium acetate, 150 mM HEPES-KOH, pH 7.4, 19 mM magnesium acetate, sterile dH_2_O) and incubated at 68°C for 15 min. Annealing and integrity of the dsRNAs were confirmed by agarose gel electrophoresis.

The dsRNA was delivered to adult worms according to the electroporation protocol of Correnti et al. [84] and Ndegwa et al. [47]. Briefly, electroporations were performed in 4 mm cuvettes with 10 couples in 50 µl electroporation buffer (Ambion) containing 25 µg dsRNA. A square-wave protocol was applied with a single 20 ms impulse at 125 V and at room temperature (Gene Pulser XCell™, Biorad). After electroporation, the worms were transferred to complete M199 medium and incubated for 5 days; 48 hours after electroporation the medium was refreshed.

### *Xenopus* oocyte transfection and inhibitor studies

Capped messenger RNA (cRNA) encoding the full-length cDNA of SmTK4 or a shortened variant containing the catalytic TK domain of SmTK4 was synthesized *in vitro* using the T7 mMessage mMachine Kit (Ambion, USA). Following synthesis, cRNA was injected into stage VI oocytes according to previously published protocols [50],[85]. To investigate Piceatannol effects, transfected oocytes were incubated with increasing concentrations (1, 2, 5, 10, 20, 50, or 100 µM) of Piceatannol. Non-injected control oocytes were incubated with the same inhibitor concentrations. As positive control progesterone was used, a steroid known to induce germinal vesicle break down (GVBD) in *Xenopus* oocytes [49]. This oocyte-specific physiological activity was detected by the appearance of a white spot at the center of the animal pole.

### *In silico* analysis

The following public domain tools were used for sequence analyses: NCBI-BLAST (http://blast.ncbi.nlm.nih.gov/Blast.cgi), the Wellcome Trust Sanger Institute *S. mansoni* OmniBlast server (http://www.sanger.ac.uk/cgi-bin/blast/submitblast/s_mansoni/omni), and GeneDB (http://www.genedb.org). For BLAST analyses to identify Syk kinases in the schistosome genome data set [6] we used the kinase domain sequence as template as the most conserved part of tyrosine kinases.

## Supporting Information

Figure S1 Influence of dsRNA treatment of *S. mansoni* couples on SmTK4-transcript level in adults. To post transcriptionally inhibit SmTK4 by RNAi, 10 worm couples each were either electroporated without dsRNA (control), with SmTK4-specific dsRNAs (1–3), or with SmTK3-specific dsRNAs (4). The amount of SmTK4 transcripts was analyzed compared to the amount of transcripts from the housekeeping gene SmPDI. In three independent experiments using SmTK4-specific dsRNAs the SmTK4 transcript level was reduced to 10–32% that of the controls. The statistical evaluation of three densitometric measurements is shown (error bars are indicated). Student's t-test (two-tailed): *p<0.001.
(1.73 MB TIF)

Table S1 Y2H interaction analyses to investigate the binding capacity of potential upstream binding partners to the tandem SH2-domain of SmTK4. Yeast cells (strain AH109) were transformed with individual prey plasmids and plasmids containing the individual N-terminal SH2(1) or C-terminal SH2(2) domain of SmTK4 (within the MCS I) and SmTK3 TK (within the MCS II), or the original bait plasmid SmTK4 SH2SH2 + SmTK3 TK pBridge, which contained both SH2 domains (tandem SH2-domain). All clones were first selected for the presence of the plasmids (Trp-/Leu-), and then for interaction (Trp-/Leu-/Ade-/His-). [+/−, growth indication: clones surviving/not surviving selection for interaction]
(0.03 MB DOC)

Table S2 GVBD assay to determine SmTK4 activity and its suppression by Piceatannol. cRNAs encoding the full-length SmTK4 cDNA or a shortened variant containing only the catalytic TK domain of this kinase were injected into stage VI oocytes of *Xenopus laevis* according to previously published protocols [50],[51],[84]. Piceatannol effects on germinal vesicle break down (GVBD; [49],[70]) were investigated by adding increasing concentrations of this inhibitor (1, 2, 5, 10, 20, 50, or 100 µM). GVBD was evaluated microscopically by the appearance of a white spot at the center of the animal pole. As negative control, non-injected oocytes were incubated with the same inhibitor concentrations. As positive control, progesterone was used, a steroid known to induce GVBD in *Xenopus* oocytes [49],[70].
(0.03 MB DOC)
