# Supplementary figures and images for: The Syk Kinase SmTK4 of Schistosoma mansoni Is Involved in the Regulation of Spermatogenesis and Oogenesis
Source: PLoS Pathog. 2010 Feb 12;6(2):e1000769. doi: 10.1371/journal.ppat.1000769 (PMC2820527; doi:10.1371/journal.ppat.1000769)

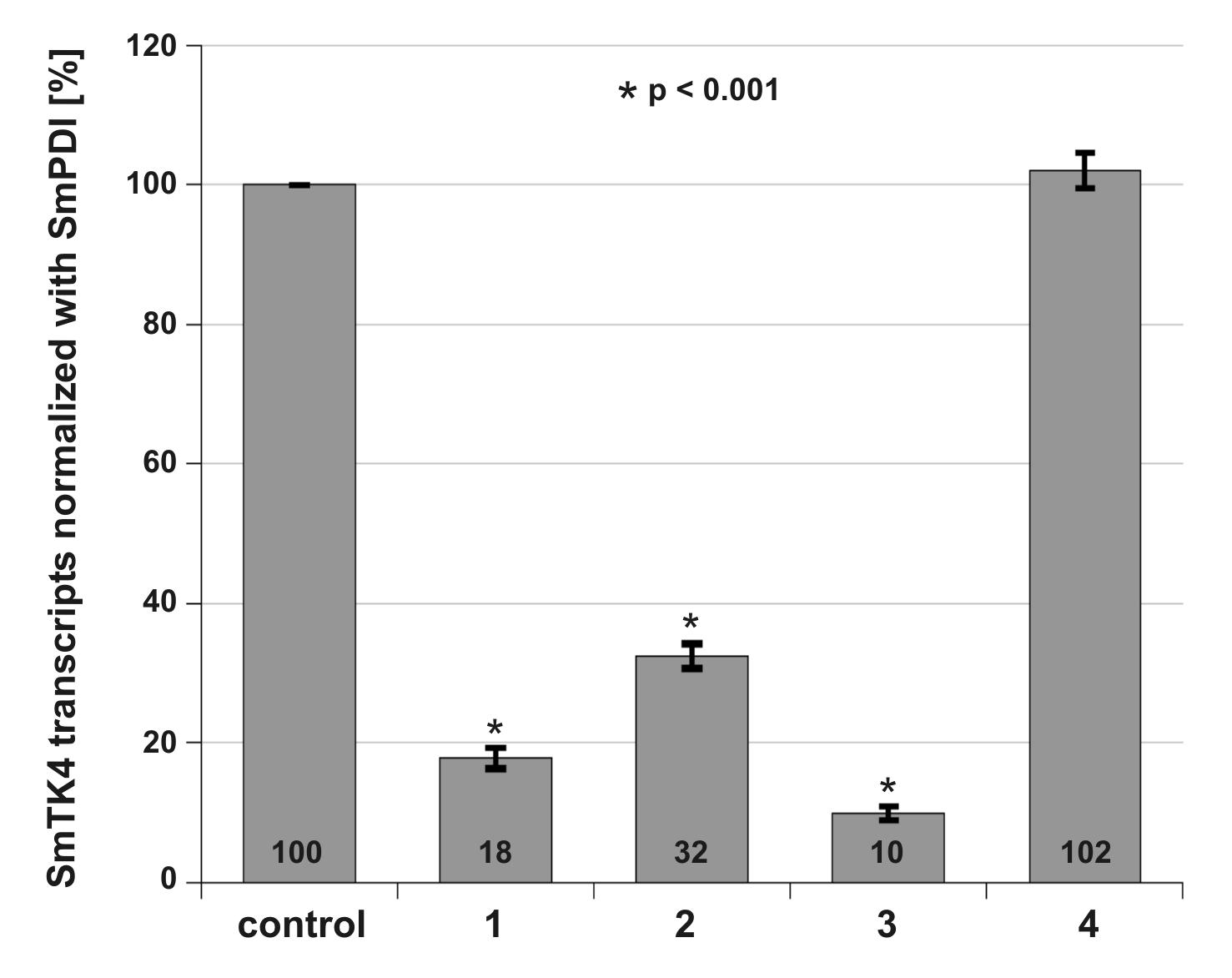

Supplement: Figure S1 — Influence of dsRNA treatment of S. mansoni couples on SmTK4-transcript level in adults. To post transcriptionally inhibit SmTK4 by RNAi, 10 worm couples each were either electroporated without dsRNA (control), with SmTK4-specific dsRNAs (1–3), or with SmTK3-specific dsRNAs (4). The amount of SmTK4 transcripts was analyzed compared to the amount of transcripts from the housekeeping gene SmPDI. In three independent experiments using SmTK4-specific dsRNAs the SmTK4 transcript level was reduced to 10–32% that of the controls. The statistical evaluation of three densitometric measurements is shown (error bars are indicated). Student's t-test (two-tailed): *p<0.001. (1.73 MB TIF) [file ppat.1000769.s001.tif]
