# Supplementary material for: The Syk Kinase SmTK4 of Schistosoma mansoni Is Involved in the Regulation of Spermatogenesis and Oogenesis
Source: PLoS Pathog. 2010 Feb 12;6(2):e1000769. doi: 10.1371/journal.ppat.1000769 (PMC2820527; doi:10.1371/journal.ppat.1000769)
Supplement: Table S1 — Y2H interaction analyses to investigate the binding capacity of potential upstream binding partners to the tandem SH2-domain of SmTK4. Yeast cells (strain AH109) were transformed with individual prey plasmids and plasmids containing the individual N-terminal SH2(1) or C-terminal SH2(2) domain of SmTK4 (within the MCS I) and SmTK3 TK (within the MCS II), or the original bait plasmid SmTK4 SH2SH2 + SmTK3 TK pBridge, which contained both SH2 domains (tandem SH2-domain). All clones were first selected for the presence of the plasmids (Trp-/Leu-), and then for interaction (Trp-/Leu-/Ade-/His-). [+/−, growth indication: clones surviving/not surviving selection for interaction] (0.03 MB DOC) [file ppat.1000769.s002.doc]

|  | SmTK4-SH2(1) + SmTK3-TK pBridge | SmTK4-SH2(2) + SmTK3-TK pBridge | SmTK4-SH2SH2 + SmTK3-TK pBridge |
| --- | --- | --- | --- |
| SmTK6 (Src kinase) | **-** | **-** | **+** |
| SmTK3 (Src kinase) | **-** | **-** | **+** |
| dipeptidyl peptidase III | **+** | **+** | **+** |
| nonsense mRNA reducing factor (NORF1) | **+** | **+** | **+** |
