# Supplementary material for: The Syk Kinase SmTK4 of Schistosoma mansoni Is Involved in the Regulation of Spermatogenesis and Oogenesis
Source: PLoS Pathog. 2010 Feb 12;6(2):e1000769. doi: 10.1371/journal.ppat.1000769 (PMC2820527; doi:10.1371/journal.ppat.1000769)
Supplement: Table S2 — GVBD assay to determine SmTK4 activity and its suppression by Piceatannol. cRNAs encoding the full-length SmTK4 cDNA or a shortened variant containing only the catalytic TK domain of this kinase were injected into stage VI oocytes of Xenopus laevis according to previously published protocols [50],[51],[84]. Piceatannol effects on germinal vesicle break down (GVBD; [49],[70]) were investigated by adding increasing concentrations of this inhibitor (1, 2, 5, 10, 20, 50, or 100 µM). GVBD was evaluated microscopically by the appearance of a white spot at the center of the animal pole. As negative control, non-injected oocytes were incubated with the same inhibitor concentrations. As positive control, progesterone was used, a steroid known to induce GVBD in Xenopus oocytes [49],[70]. (0.03 MB DOC) [file ppat.1000769.s003.doc]

| **Piceatannol [µM]** | **SmTK4 full-length** | **SmTK4-TK** | **control** | **control + progestrone** |
| --- | --- | --- | --- | --- |
| **0** | 0 | 100 | 0 | 100 |
| **1** | 0 | 100 | 0 | 100 |
| **2** | 0 | 62 | 0 | 100 |
| **5** | 0 | 0 | 0 | 100 |
| **10** | 0 | 0 | 0 | 100 |
| **20** | 0 | 0 | 0 | 100 |
| **50** | 0 | 0 | 0 | 100 |
| **100** | 0 | 0 | 0 | 100 |
